# An integrated assessment of land use impact, riparian vegetation and lithologic variation on streambank stability in a peri-urban watershed (Nigeria)

**DOI:** 10.1038/s41598-022-15008-w

**Published:** 2022-06-29

**Authors:** Chukwueloka A. U. Okeke, Jonathan Uno, Sunday Academe, PraiseGod Chidozie Emenike, Tamunoene K. S. Abam, David Olugbenga Omole

**Affiliations:** 1 Department of Earth Sciences, Anchor University Lagos, 1 – 4 Ayobo Road, Lagos, Nigeria; 2 Civil, Infrastructure and Environment Research Cluster, Department of Civil Engineering, Covenant University, Ota, Ogun State Nigeria; 3 Institute of Geosciences and Space Technology, Rivers State University, Nkpolu – Oroworukwo, P.M.B. 5080, Port Harcourt, Nigeria

**Keywords:** Ecology, Environmental sciences, Natural hazards

## Abstract

Bank erosion is an important source of sediment and phosphorus to inland fluvial systems and is generally responsible for more than half of the total watershed sediment export. Numerous studies have quantified bank erosion and the spatio-temporal variation of sediment flux in different watersheds. However, there is sparse research to date on the linkages between bank erosion/accretion and sediment export under different land uses, especially in rapidly evolving peri-urban watersheds. This research, therefore, integrated remote sensing techniques and repeated field survey of erosion pin plots to quantify bank erosion and sediment flux in the 80 km^2^ Nkisi River watershed (NRW), southeast Nigeria, over a three-year period. The impact of land use change on streambank erosion was evaluated by utilising remotely sensed Landsat datasets of 2003, 2010, 2016 and 2019. Geotechnical tests were used to characterise the lithologic properties of the banks, while the Bank Stability and Toe Erosion Model (BSTEM) was used to determine the stability of the banks under various hydrological conditions and mechanical properties of the riparian vegetation. Mean bank recession rates increased from 10.7 cm during the 2017–2018 monitoring period to 17.5 cm for the 2019–2020 monitoring period. The percentage of total watershed export ascribed to bank erosion in the three stream reaches varied from 6.6 to 44.9%. The high rates of bank erosion and accretion within the NRW were attributed to rapid changes in land use, which evolved from grassland and woodland to cropland, built-up and bare land. The BSTEM accurately predicted the high erosion rates of the streambanks and showed that riparian vegetation has a mechanical effect on bank stability. However, the mechanical effect diminishes as the depth to water table rises during high streamflow events.

## Introduction

Streambank erosion and channel planform dynamics are two important hydrogeomorphic processes that play a significant role in the evolution of fluvial landforms^1–3^. Large sediment pulses generated by hillslope processes, bank incision and channel migration have been considered a potential threat to the aquatic and terrestrial biota^4,5^. As a result, the high concentration of heavy metals and nutrients in streams and river sediments, which occur via accelerated bank erosion and hillslope processes in many fluvial systems, has been attributed to an increase in urbanisation and intensified agricultural activities^4,6,7^. Therefore, an understanding of the relationships between sediment supply and hillslope-channel coupling in a rapidly evolving peri-urban watershed is essential for the effective quantification of sediment loads to river channels and the development of sustainable river management strategies. It is noteworthy to mention that some investigators have conducted research on the contributory mechanisms of streambank instability^8,9^. However, the complex relationship between the rate of bank erosion and accretion in small peri-urban watersheds has not been fully understood due to the spatiotemporal variations in climate and hydrologic regimes.

Reports have revealed that land use-induced alterations depend on the level of hydrological and sedimentological connectivity between the hillslopes and stream channels, causing anthropogenic activities, such as sand mining, deforestation, channelisation, dam construction and numerous river training works to impact significant changes in the hydrologic response of watersheds^10,11^. To this end, the timing and magnitude of runoff to streams and river channels gets modified. Meanwhile, numerous studies have evaluated the impacts of land use change and climate variability on the rate of bank erosion and accretion in some of the world’s deltaic landscapes, such as the Niger Delta region^12^, the Mekong River Delta^13,14^, and the Yangtze River Delta^15^. In corroboration, Royall et al.^16^ and Johnson and Royall^17^ observed that urbanisation contributes to the alteration of stream channels’ dynamic equilibrium by modifying their response to hydrological and meteorological variables. The outcome of the stream channel modification increases the frequency and magnitude of flood discharge that ultimately leads to bank erosion, channel incision and aggradation^16,17^, whereas rapid changes in land use patterns alter the response of streams and river channels to hydrogeomorphic disturbance regimes^18,19^. However, there is sparse research to date on the linkages between bank erosion and accretion rates, especially in small, rapidly evolving peri-urban watersheds^67^.

Streambank retreat occurs through the combination of two dominant mechanisms: (1) basal erosion of in situ bank toe material by hydraulic shear stress and (2) geotechnical failure of the upper parts of the bank due to the effect of gravitational forces^20,21^. Generally, the stability of streambanks is primarily controlled by the ratio between the resisting forces of the bank and the driving forces. The resisting forces are a function of the shear strength of the bank material, while the driving forces are controlled by soil weight, antecedent soil moisture, and bank geometry. Furthermore, riparian vegetation and lithologic characteristics are two important factors controlling the stability of streambanks^22,23,67^. In a similar vein, Konsoer et al.^24^, mentioned that lithologic characteristics influence the rate of bank failure and determine the characteristic failure mechanisms of streambanks. Furthermore, they pointed out that the grain-size distribution and percentage of clay-sized sediments determine the cohesion and resistance of in situ bank materials to hydraulic shear stress.

The influence of riparian vegetation on the stability of streambanks has been divided into hydrological and mechanical effects^25,26^. The mechanical effect of riparian vegetation occurs via root-reinforcement that increases the shear strength of the composite bank material as a result of root tensile strength, areal density and distortion. On the other hand, the hydrological effect of vegetation occurs via transpiration which reduces soil moisture and interception of rainfall that minimises direct infiltration into the bank^27^. These two processes reduce positive pore-water pressure and enhance the shear strength of the bank material, thereby increasing the stability of the streambank. However, several researchers have noted that the stability of streambanks depends on the type of vegetation and the nature of agricultural activities within the riparian area^18,28^. Simon and Collinson^27^ and Yu et al.^29^ analysed the hydrological and mechanical effects of different plant species on bank stability and observed that root cohesion varied across the plant species due to their different tensile strength and root area ratios.

Numerous techniques have been used to estimate streambank erosion rates, including the utilisation of geographic information systems (GIS) and remote sensing (RS), aerial photographs, erosion pins, terrestrial laser scanners, and repeated bank-line surveys^30,31^. Erosion pins and repeated bank-line surveys are two traditional methods of measuring bank erosion. Erosion pins are mostly preferred over other methods because the technique is cost-effective, accurate and unsophisticated, and thus can be used in developing countries to reduce the high cost of replacing expensive monitoring workstations installed in remote areas. Erosion pins have been generally integrated with other methods such as total station, terrestrial laser scanners, aerial photogrammetry, and artificial neural networks to quantify bank erosion rates in many watersheds^32,33^. Therefore the objectives of this research were to (1) characterise the various land use and land cover (LULC) types and land use change patterns in the watershed from 2003 to 2019 and analyse their connectivity with bank erosion and channel instability, (2) evaluate the influence of riparian vegetation and lithologic characteristics on bank erosion in the watershed, and (3) determine the recession rates of the streambanks and quantify the total percentage of watershed sediment exported to the river from 2017 to 2020.

## Methods

### Study area

**Figure 1:**
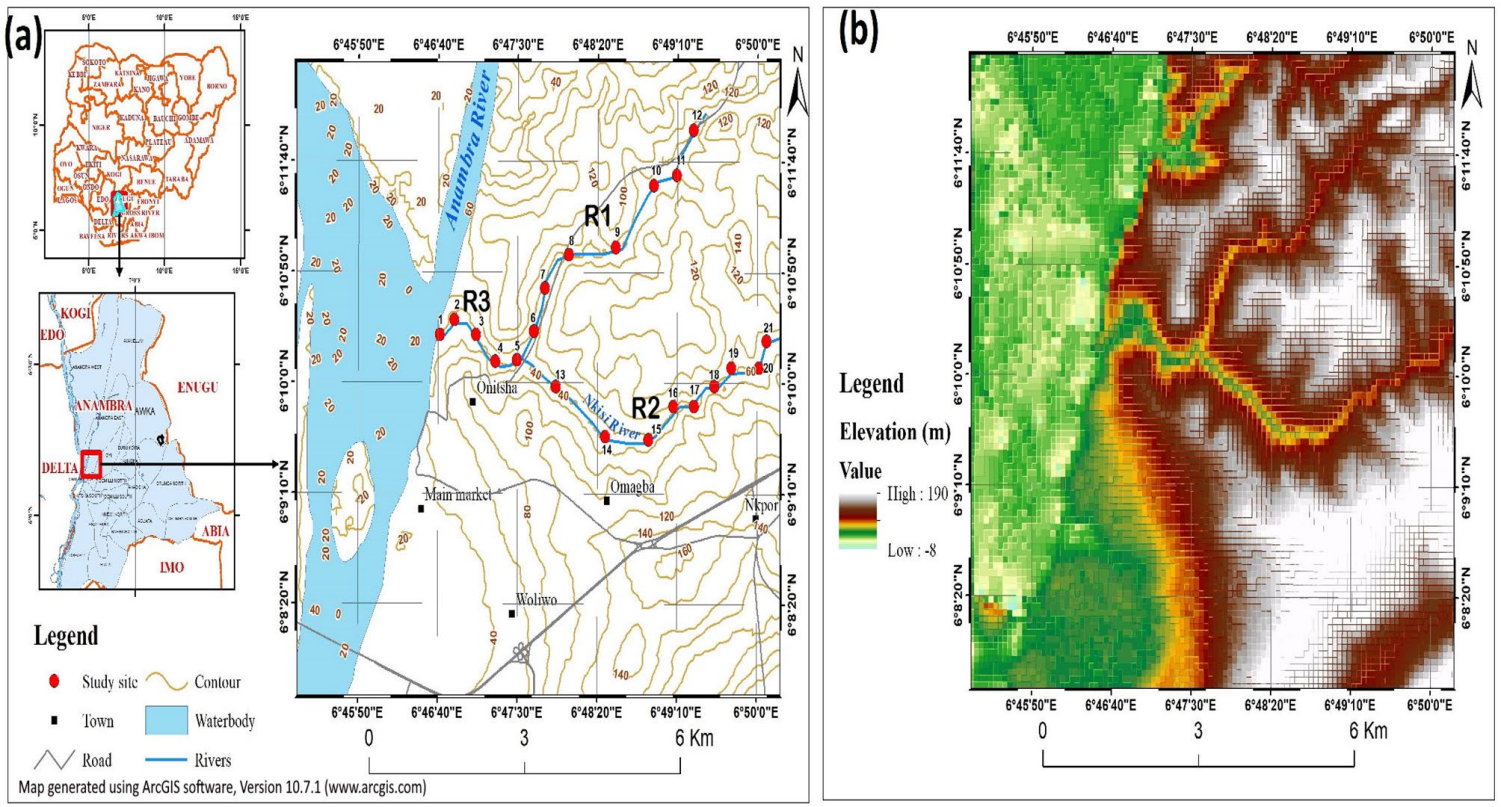
(**a**) Location of Nkisi River watershed in Anambra State (Nigeria) showing the study sites and (**b**) the digital elevation model of the study area. *R1*, *R2* and *R3* represent *Reach 1*, *Reach 2* and *Reach 3*, respectively. The figure was generated by using an ArcGIS software (version 10.7.1). One of the spatial analysis tools (contour tool) in the ArcGIS software was used to generate contour lines from a 30-m DEM image of the study area which was downloaded from the United States Geological Survey website (https://earthexplorer.usgs.gov/). The study locations overlying the map was obtained from the field using a GPS device (model no: Garmin eTrex 10).

The Nkisi River watershed (NRW) is located on the eastern bank of the Niger River, in Onitsha, Anambra State, Nigeria (Fig. 1a). The river has two main tributaries, Nkwelle Ezunaka and Nkpor-Ogbunike streams, which originate from the north–south trending Awka-Umuchu-Orlu cuesta and merge in the northern outskirts of Onitsha before debouching into the Niger River. The watershed elevation ranges from 0 (sea level) to 190 m (Fig. 1b) and lies within the transition zone between the subequatorial climate and the hinterland climatic belts of Nigeria. The river drains approximately 80 km^2^ of NRW and receives an average annual precipitation of 1850 mm. The watershed is bounded to the north by the Anambra (Omambala) River basin, to the south by the Idemili River basin, to the east and west by the Awka-Umuchu-Orlu cuesta and the Niger River, respectively.

The dominant geologic units within the watershed are the Paleocene Imo Shale and the Eocene Ameki Group. The Imo Shale outcrops in the lower reaches of the river channel and consists of well indurated and highly resistant mudstone, which make up the basal layers of the streambanks. The middle and upper sections of the watershed are dominated by the westerly dipping hillslopes of the Awka-Umuchu-Orlu escarpment, which are composed of erodible and ferruginous sediments of the Ameki Group. The eastern part of the watershed is characterised by fluvially dissected hilly terrain, while the western part is dominated by the alluvial plains of the Lower Niger region. The channel bed slope is typically high at the headwaters of the stream (0.03 ~ 0.05) and relatively low near the river mouth (0.004). The majority of the watershed area is dominated by a tropical forest landscape that has been extensively degraded in the past three decades due to an increase in urbanisation. The hydrologic regime of the river is typically perennial with a flashy hydrograph, especially during the peak rainfall months of June and September. Sediments in the upper and middle reaches of the riverbed comprise poorly sorted silty clay with occasional gravel that changes to well-sorted gravelly sand containing less than 10% fines at the river mouth. The upper reaches of the river channel are deeply incised and have been undergoing accelerated bank erosion due to numerous anthropogenic stresses, including deforestation, sand mining, agricultural intensification (row crop farming), and rapid urbanisation.

### Soil sampling and geotechnical analysis

A total of 47 undisturbed soil cores were recovered from the study area using standard U4 soil samplers. Two representative soil cores were recovered along a vertical section at each of the study sites in *Reach 1* and *Reach 2*, respectively. In contrast, three representative soil cores were recovered along a vertical axis in all the 5 study sites in *Reach 3*. The soil samples were obtained at a vertical spacing that ranged from 0.5 to 3 m depending on bank height. Similarly, 21 soil samples were collected from the talus materials at the bank toe to evaluate changes in their geotechnical and sedimentological properties. Lithologic characterisation of the streambanks was achieved by subjecting the soils to geotechnical tests, such as the Atterberg limits test, grain-size distribution analysis, unconsolidated undrained (UU) triaxial test, and phase relationship tests. All the tests were carried out following specific ASTM standards. Further information on the engineering behaviour of the bank materials was obtained by classifying the soils using the Unified Soil Classification System (USCS), which utilised the results obtained from the soils’ index properties and plasticity.

### Land use/land cover (LULC) assessment

The LULC classification was carried out in two phases: *phase I* and *phase II*. The first phase was carried out to assess the LULC dynamics of the entire watershed. In contrast, the second phase, which focused primarily on the lower regions of the watershed (*Reach 3*), assessed the rates of streambank erosion and deposition, channel widening, accretion and destruction of the riparian corridor. In *phase I*, multi-temporal datasets made up of Landsat-7 Enhanced Thematic Mapper (ETM) imageries of 2003, 2010, and Landsat-8 Operational Land Imager (OLI) of 2016 and 2019 with a spatial resolution of 30 m were used for the LULC identification and classification (Table 1a). The Landsat imageries, with path 189 and row 56, were downloaded from the United States Geological Survey—earth explorer website (https://earthexplorer.usgs.gov/). The watershed boundary was delineated by using a Shuttle Radar Topographic Mission (SRTM) Digital Elevation Model (DEM) with a spatial resolution of 30 m, while the topographic maps (1:50,000) of 1997 and 2017 were used to acquire additional information regarding the study area and the entire watershed. Ground truthing was also carried out from November 2017 to June 2019 using a portable Global Positioning System (GPS—model no: Garmin eTrex 10). The ground truth data, which represent reference data points, were used for geometrical correction, supervised classification and overall accuracy assessment.

**Table 1:** Datasets used for the LULC analysis for (a) the entire study area and (b) *Reach 3* area.

Data type	Year of acquisition	Spatial resolution	Format	Source
(a)				
Landsat image (ETM) 07	2003	30 m	Raster	USGS
Landsat image (ETM) 07	2010	30 m	Raster	USGS
Landsat image (OLI) 08	2019	30 m	Raster	USGS
Topographic maps/SRTM base maps	1997 and 2007	1:50,000/30 m	Raster	FSN
(b)				
Landsat image (TM) 05	2003	30 m	Raster	USGS
Landsat image (OLI) 08	2016	30 m	Raster	USGS
Landsat image (OLI) 08	2019	30 m	Raster	USGS
Topographic maps	1997 and 2007	1:50,000	Raster	FSN

*USGS* United States Geological Survey, *FSN* Federal Surveys of Nigeria.

The satellite data were preprocessed by importing them into ArcGIS 10.7.1 for geo-referencing, mosaicking and sub-setting of the images based on the Area of Interest (AOI). Atmospheric and radiometric corrections were performed on the satellite data by using the Dark Object Subtraction (DOS) method and by converting the raw digital numbers (DNs) to top-of-atmosphere (TOA) reflectance. The remotely sensed data were geo-rectified by identifying and specifying the actual coordinates of about twenty evenly distributed ground control points (GCPs), while a polynomial function was used to fit the coordinates of the raw image to that of the GCPs. The georectified aerial photographs showed root mean square error (RMSE) values that were less than 0.5, which indicates a high georectification accuracy. Image enhancement was carried out using the contrast stretching technique to adjust the histogram and redistribute the image pixels. The Landsat imageries were further examined by allotting per-pixel signatures, while the specific DN value of the various terrain elements was used to classify the watershed into five representative land use classes: built-up area, thick vegetation, light vegetation, sand deposit/floodplain, and water body. The Maximum Likelihood Classification (MLC) algorithm was used to carry out a supervised classification of the images. The MLC algorithm is one of the most conventional supervised classification methods which assigns a pixel to a class with the highest likelihood by utilising a discriminant function. Accuracy assessment was performed using the equalised stratified random sampling method wherein about 200 training points were randomly distributed to represent the different LULC classes of the watershed. The relative relationship between the classification results and the reference data was quantitatively evaluated using the confusion matrix, while the kappa test was performed to assess the degree of accuracy of the classification results.

In *phase II*, LULC classification was carried out using Landsat-5 ETM and Landsat-8 OLI imageries with a spatial resolution of 30 m, in addition to topographic maps (1:50,000) and Google Earth images of 2003, 2016, and 2019 (Table 1b). The satellite data were preprocessed and classified following the methods utilised in *phase I*. The LULC maps were categorised into seven classes such as built-up, floodplain, light vegetation, thick vegetation, riparian vegetation, sand deposit, and water body. Accuracy assessment was also performed using the equalised stratified random sampling method.

### The Bank Stability and Toe Erosion Model (BSTEM)

The Bank Stability and Toe Erosion Model (BSTEM) is one of the most commonly utilised process-based numerical models for predicting the stability of streambanks. The BSTEM was developed by the National Sedimentation Laboratory, Oxford, Mississippi (USA), and has been adjudged by several researchers as one of the most advanced process-based models for predicting bank stability and channel adjustments in alluvial channels^34^. The BSTEM has been utilised to dynamically evaluate the stability of banks under various hydraulic, hydrologic, and geotechnical conditions^35^. The current version of the model (BSTEM Static Version 5.4) comprises the bank stability submodel and the toe erosion submodel. The major input parameters required for the BSTEM include bank geometry, soil strength parameters, bed slope, stream stage, and groundwater level. The toe erosion submodel of the BSTEM estimates fluvial undercutting of the bank by hydraulic shear stresses^20^. The submodel uses the channel geometry and flow parameters of the stream to estimate the average boundary shear stress acting on each node of the bank as defined by

1 $$\tau_{o} = \gamma_{w} RS$$

where $\tau_{o}$ = average boundary shear stress (Pa), $\gamma_{w}$ = unit weight of water (9.81 ${\text{kN}}/{\text{m}}^{3}$), $R$ = local hydraulic radius (m), and $S$ = channel slope (m/m). The submodel also utilises the excess shear stress equation proposed by Partheniades^36^ to estimate the mean erosion rate for each node:

2 $$\varepsilon = k_{d} \left( {\tau_{o} - \tau_{c} } \right)^{a}$$

where $\varepsilon$ = erosion rate (m/s), $k_{d}$ = erodibility coefficient (${\text{m}}^{3} \,{\text{N}}^{ - 1} \,{\text{s}}^{ - 1}$), $\tau_{c}$ = critical shear stress (kPa), and $a$ = exponent normally assumed to be unity.

The bank stability module of the BSTEM determines the stability of streambanks by computing a factor of safety ($FoS$) as the ratio of the resisting forces to the driving forces, utilising three limit equilibrium-method models: horizontal layers, vertical slices, and cantilever shear failures. The resisting forces in the model are defined by the Mohr–Coulomb failure criterion for the saturated zone of the failure plane, which can be expressed as

3 $$s_{r} = c^{\prime} + \left( {\sigma - \mu_{w} } \right)\tan \varphi^{\prime}$$

where $s_{r}$ = shear strength of the soil (kPa), $c^{\prime}$ = effective cohesion (kPa), $\sigma$ = normal stress (kPa), $\mu_{w}$ = pore-water pressure (kPa), and $\varphi^{\prime}$ = effective friction angle (degrees). Additionally, the model considers the influence of negative pore-water pressure (matric suction) in increasing soil cohesion for the unsaturated zone of the failure plane by incorporating the modified Mohr–Coulomb failure criterion proposed by Fredlund et al.^37^:

4 $$s_{r} = c^{\prime} + \left( {\sigma - \mu_{a} } \right)\tan \varphi^{\prime} + \left( {\mu_{a} - \mu_{w} } \right)\tan \varphi^{b}$$

where $\left( {\sigma - \mu_{a} } \right)$ = net normal stress on the failure plane at failure, $\left( {\mu_{a} - \mu_{w} } \right)$ = matric suction (kPa), and $\mu_{a}$ = pore-air pressure (kPa), and $\varphi^{b}$ ranges from 10° to 20° depending on soil type. The driving force is defined by

5 $$s_{d} = W\sin \left( \beta \right)$$

where $s_{d}$ = driving stress (kPa), $W$ = weight of the soil (wet) block per unit area of failure plane (kN/m^2^), and $\beta$ = angle of the failure plane (degrees). The BSTEM iteratively computes the minimum $FoS$ by considering different combinations of shear emergence elevation on the bank face and failure plane angle.

The BSTEM has been extensively used to predict bank stability and sediment loading in many fluvial systems by considering the influence of various geotechnical and geomorphological factors, such as lithologic characteristics, riparian vegetation, water table condition, flow dynamics of the river, and bank geometry. However, little or no research has been done to compare the results obtained from conventional methods of measuring bank erosion (e.g., erosion pins and repeated bank-line survey) with a process-based model such as the BSTEM.

### Bank erosion monitoring and hydrogeomorphological analysis

Monitoring of bank erosion and river channel dynamics were carried out at approximately 21 study sites within the lower and upper reaches of the river. The survey was carried out at quarterly intervals from June 2017 to December 2020 using two traditional field survey methods, such as repeated cross-profiling of the river channel and measurement of rates of bank erosion using erosion pins of length and diameter 1 m and 12 mm, respectively. Additional data on the nature of the river channel were collected by utilising the visual assessment protocol developed by the Natural Resources Conservation Services of the United States Department of Agriculture (USDA-NRCS, 2019). The geometric variables measured include bank height, bank angle, channel width, bed slope, and thalweg location. The entire length of the river was carefully measured and divided into three reaches: *Reach 1*—Nkwelle Ezunaka reach (*R1*), *Reach 2*—Nkpor-Ogbunike reach (*R2*), and *Reach 3*—Lower Nkisi River reach (*R3*) (see Fig. 1). The 21 study sites were mostly located at channel bends, where active bank erosion has been observed. About 116 erosion pins were installed horizontally at the lower and upper reaches of the river following the design method adopted by Myers et al.^38^ and Casagli et al.^39^. The accuracy of the erosion monitoring data at each study site in *Reach 1* and *Reach 2* was improved by installing six (6) erosion pins along a vertical section with a (vertical) pin spacing that varied from 0.5 to 1 m. During the first two years (June 2017–May 2019), data collection and measurement of bank erosion were carried out at each of the study sites on a monthly basis, especially after major stormflow events, whereas twelve (12) rounds of measurements were made between June 2019 and December 2020. Nkisi River is an ungauged stream; hence monthly discharge was estimated from Manning’s equation by utilising the monthly hydrometric data obtained during the field survey as shown in Eq. (6)

6 $$Q = A\left( {R^{2/3} S^{1/2} } \right)/n$$

where $A$ = cross-sectional area of the stream channel (m^2^), $R$ = hydraulic radius (m), and $n$ = Manning’s roughness coefficient. Suspended sediment concentration was determined by utilising the grab sampling technique. Total suspended sediment load at each stream reach was estimated by multiplying the mean monthly discharge (*Q* [m^3^/s]) by the mean monthly sediment concentration (*SSC* [mg/L]), while the rate of streambank sediment loss per year ($s_{L}$) at each stream reach was estimated by using the equation below^40,41^:

7 $$s_{L} = BH \times BL \times \delta \times \gamma$$

where $s_{L}$ = rate of streambank sediment loss (t/yr), $BH$ = bank height (m), $BL$ = reach length (m), $\delta$ = recession rate (m/yr), and $\gamma$ = soil bulk density (Mg/m^3^). The recession rate at each of the study site was also estimated by dividing the total eroded area in a given period by the total number of erosion pins used.

### Bank stability analysis and modelling conditions

Bank stability analysis was conducted by incorporating the geometrical characteristics of the streambanks into the BSTEM, in addition to using the soil strength parameters ($c^{\prime}$ and $\varphi^{\prime}$), the default erosion resistance parameters ($k_{d}$ and $\tau_{c}$), and the stream stage at each stream reach measured after major hydrologic events. Furthermore, the influence of riparian vegetation on the stability of the banks was evaluated by incorporating the root-reinforcement submodel (RipRoot) in the BSTEM. RipRoot represents a load-sharing fibre-bundle model which typically simulates the gradual breaking of plant roots within the soil matrix and subsequently computes the minimum applied load for root breakage^42^. The model also computes additional cohesion due to plant roots ($c_{r}$) using the plant age and percent contribution to assemblage, as proposed by Abernethy^26^, Simon and Collison^27^ and Pollen-Bankhead and Simon^43^. Hence, Eq. (3) can be modified as follows:

8 $$s_{sr} = c^{\prime} + c_{r} + \left( {\sigma - \mu_{w} } \right)\tan \varphi^{\prime}$$

where $s_{sr}$ = shear strength of the soil-root matrix (kPa).

This research adopted two model conditions by considering the relationship between the stream stage and groundwater table and their influence on bank stability. The first series of BSTEM simulations evaluated the stability of *Reach 1* and *Reach 2* streambanks under two hydrologic conditions: low and high stream stage scenarios, with a corresponding flow elevation of 1 and 5 m, respectively. For each of the stream stages, bank stability analysis was carried out with respect to the depth to groundwater table ($GWT_{depth}$), which was varied from 1 to 5 m. The second series of BSTEM simulations evaluated the stability of *Reach 3* streambanks by considering the influence of riparian vegetation on bank retreat at groundwater table depths of 1.8 and 2.2 m, respectively. The dominant plant species within the riparian corridor and their percent distribution are *Pennisetum purpureum* (80%), *Andropogon gayanus* (10%), *Acroceras amplectens* (5%), and *Amaranthus spp.* (5%). However, the stability analysis was performed by relating the riparian area to wet meadow vegetation with a plant age of 5 years and 100% contribution to assemblage. The default parameters in the root-reinforcement submodel were used in the simulation due to the limitations associated with determining the root area ratio, root diameter, and tensile strength of different plant species within the riparian corridor. Consequently, the $c_{r}$ values obtained at different rooting depths using the RipRoot submodel were utilised to evaluate the stability of the banks, as summarised in Table 2.

**Table 2:** Parameters used for the bank stability analysis using the BSTEM.

$GWT_{depth}$ = 1.8 m	Root depth (m)	**0**	**0.2**	**0.5**	**0.7**	**1.0**
	$c_{r}$(kPa)	0	17.6	6.9	4.9	3.5
$GWT_{depth}$ = 2.2 m	Root depth (m)	**0**	**0.2**	**0.5**	**0.7**	**1.0**
	$c_{r}$(kPa)	0	17.7	7.1	5.1	3.5

$c_{r}$ = root cohesion, $GWT_{depth}$ = depth to groundwater table.

Significant values are in bold.

## Results

### Dynamics of LULC change in NRW

**Figure 2:**
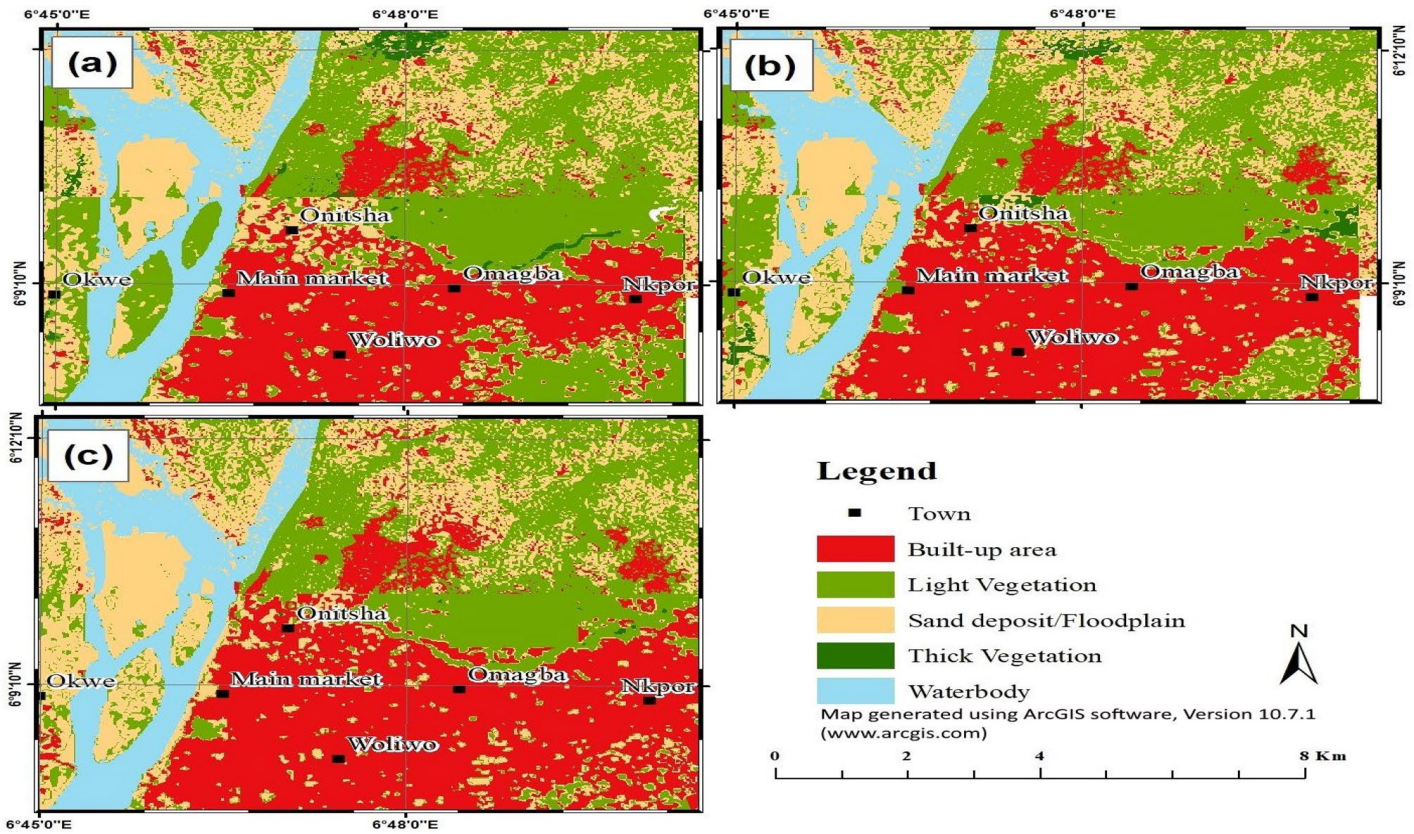
Land use/land cover (LULC) maps of the Nkisi River watershed for (**a**) 2003 (**b**) 2016 and (**c**) 2019. The LULC analysis was carried out using Landsat 7 ETM+ and Landsat 8 OLI satellite imagery of 2003, 2010 and 2019 in an ArcGIS software (version 10.7.1). Supervised image classification method was carried out by utilising the training sites developed from a Google earth application. An overlay analysis in an ArcGIS software was performed to determine the changes in LULC between the reference periods. Further information on the LULC classification maps of the study area can be found via the link in the supplementary data.

Table 3 and Fig. 2 present the spatial and quantitative distribution of LULC categories for the three reference years. The overall classification accuracy was 89% for the 2003 LULC map, 94.2% for the 2010 LULC map, and 93.8% 2019 LULC map. These classification accuracies fall within and slightly above the standard range of 85–90%^44,45^. Additionally, the image classification results produced an overall kappa coefficient of 0.86 for 2003, 0.91 for 2010 and 0.89 for 2019. The LULC classification results show that built-up, light vegetation and sand deposit/floodplain were the most dominant LULC classes throughout the study period. A detailed analysis of the LULC maps shows that in 2003, light vegetation (3803 Ha) and sand deposit/floodplain (2619 Ha) dominated the northern parts of the study area, while built-up (2497 Ha) dominated the densely populated southern region of the study area (Onitsha city centre). However, in 2010, light and thick vegetation and water body decreased considerably while sand deposit/floodplain and built-up increased by 3.2 and 22%, respectively. A similar trend of LULC dynamics was observed in 2019, with light and thick vegetation decreasing by 15.5 and 91.5%, respectively, at the expense of built-up and sand deposit/floodplain. In general, the trend of the results reveals that water body and light and thick vegetation decreased throughout the study period due to unregulated urban sprawl and other anthropogenic activities that led to the degradation of the peri-urban landscape.

**Table 3:** Land use/land cover change in the watershed from 2003 to 2019.

Land use class	Area (Ha)			%			Area difference (Ha)		
	2003	2010	2019	2003	2010	2019	2003–2010	2010–2019	2003–2019
Built-up area	2497	3044	3388	24.23	29.53	32.87	547	344	891
Light vegetation	3803	3205	2709	36.90	31.10	26.28	− 598	− 496	− 1094
Thick vegetation	130	117	10	1.26	1.13	0.10	− 13	− 107	− 120
Sand deposit/floodplain	2619	2702	2988	25.41	26.22	28.99	83	286	369
Water body	1258	1239	1213	12.21	12.02	11.77	− 19	− 26	− 45
Total	10,307	10,307	10,308	100	100	100			
Accuracy assessment	86.96	94.80	92.20						
Kappa statistics	0.86	0.91	0.89

**Figure 3:**
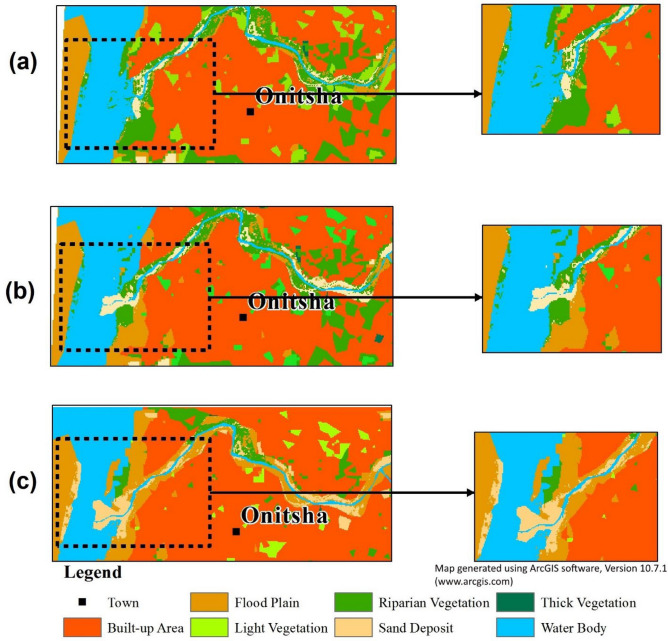
Trends of land use/land cover (LULC) change in *Reach 3* area for (**a**) 2003 (**b**) 2016 and (**c**) 2019. By using an ArcGIS software (version 10.7.1), the LULC maps were obtained by producing a detailed subset of Fig. 2 (*Reach 3* area) to clearly examine the rates of bank erosion, channel widening and accretion under different land uses.

Table 4 and Fig. 3 show the effects of LULC change on the rate of accretion/sedimentation and morphology of the river channel. The classification results show a general increase of built-up, floodplain and sand deposits throughout the study period, while vegetation (riparian, light and thick) and water body area decreased considerably during the same period. From 2003 to 2019, riparian vegetation, water body, light and thick vegetation all decreased by 60.6, 7.4, 60.8, and 89.2%, respectively. In contrast, sand deposits, floodplain, and built-up increased by 148.4, 78.2, and 13.2%, respectively. The highest period of channel width expansion was recorded during the 2016–2019 monitoring period, which coincided with the peak period of riparian forest destruction that led to bank erosion and accretion.

**Table 4:** Land use/land cover change in the *Reach 3* area from 2003 to 2019.

Land use class	Area (Ha)			%			Area difference (Ha)		
	2003	2016	2019	2003	2016	2019	2003–2016	2016–2019	2003–2019
Built-up area	178.67	191.43	202.18	49.35	52.88	55.85	12.76	10.75	23.51
Floodplain	31.02	42.38	55.28	8.57	11.71	15.27	11.36	12.90	24.26
Light vegetation	34.14	15.35	13.39	9.43	4.24	3.70	− 18.79	− 1.95	− 20.74
Thick vegetation	1.11	0.12	0.12	0.31	0.03	0.03	− 1.00	0.00	− 1.00
Riparian vegetation	57.08	46.13	22.47	15.77	12.74	6.21	− 10.95	− 23.66	− 34.61
Water body	51.67	51.05	47.86	14.27	14.10	13.22	− 0.62	− 3.19	− 3.81
Sand deposit	8.34	15.56	20.72	2.30	4.30	5.72	5.72	7.23	12.38
Total	362.02	362.02	362.02	100	100	100			
Accuracy assessment	86.96	94.80	92.20

### Streambank erosion rates and total annual sediment loads

**Figure 4:**
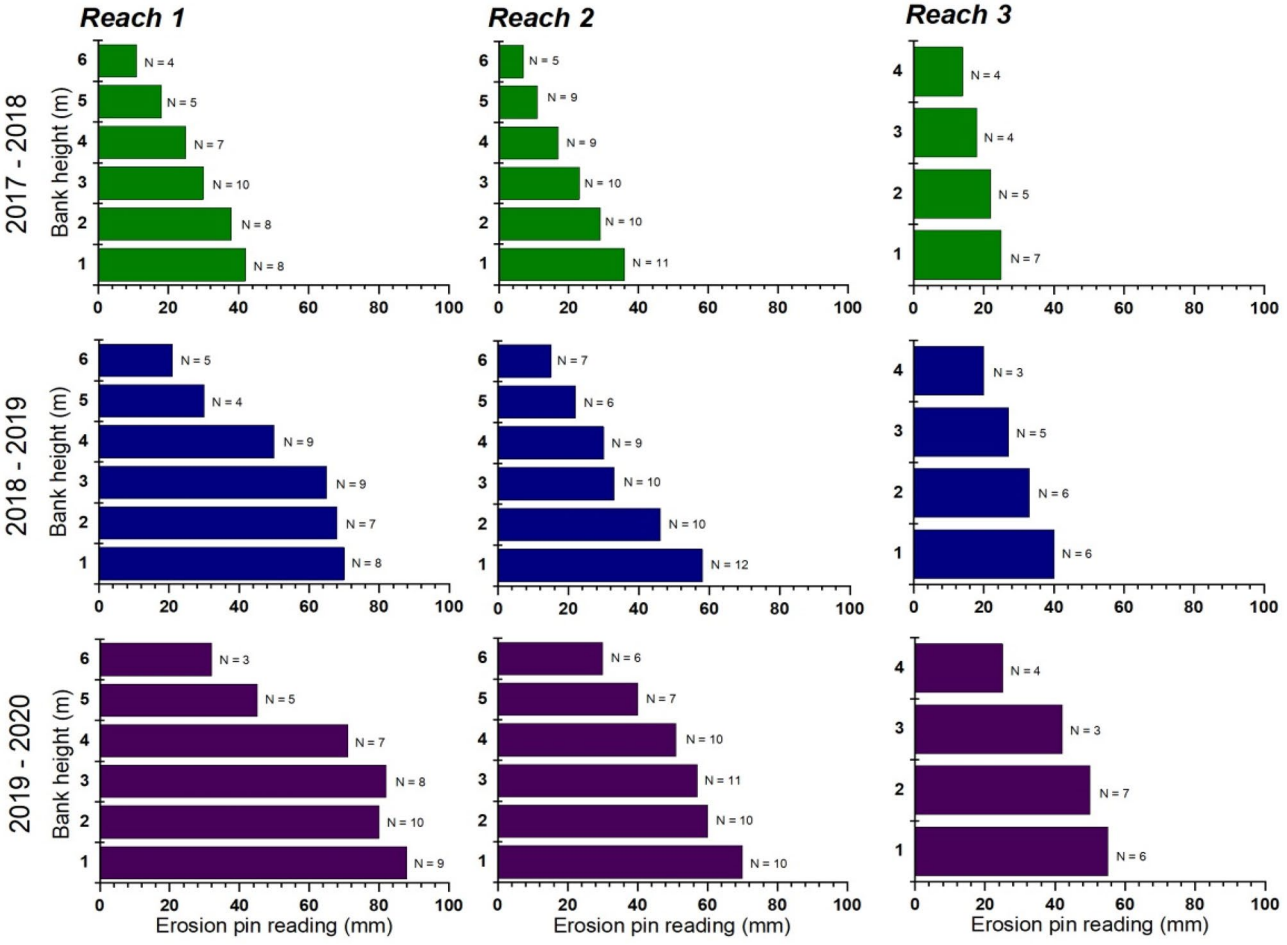
Mean erosion pin readings measured from the exposed bank faces within the three study reaches from June 2017 to December 2020.

**Figure 5:**
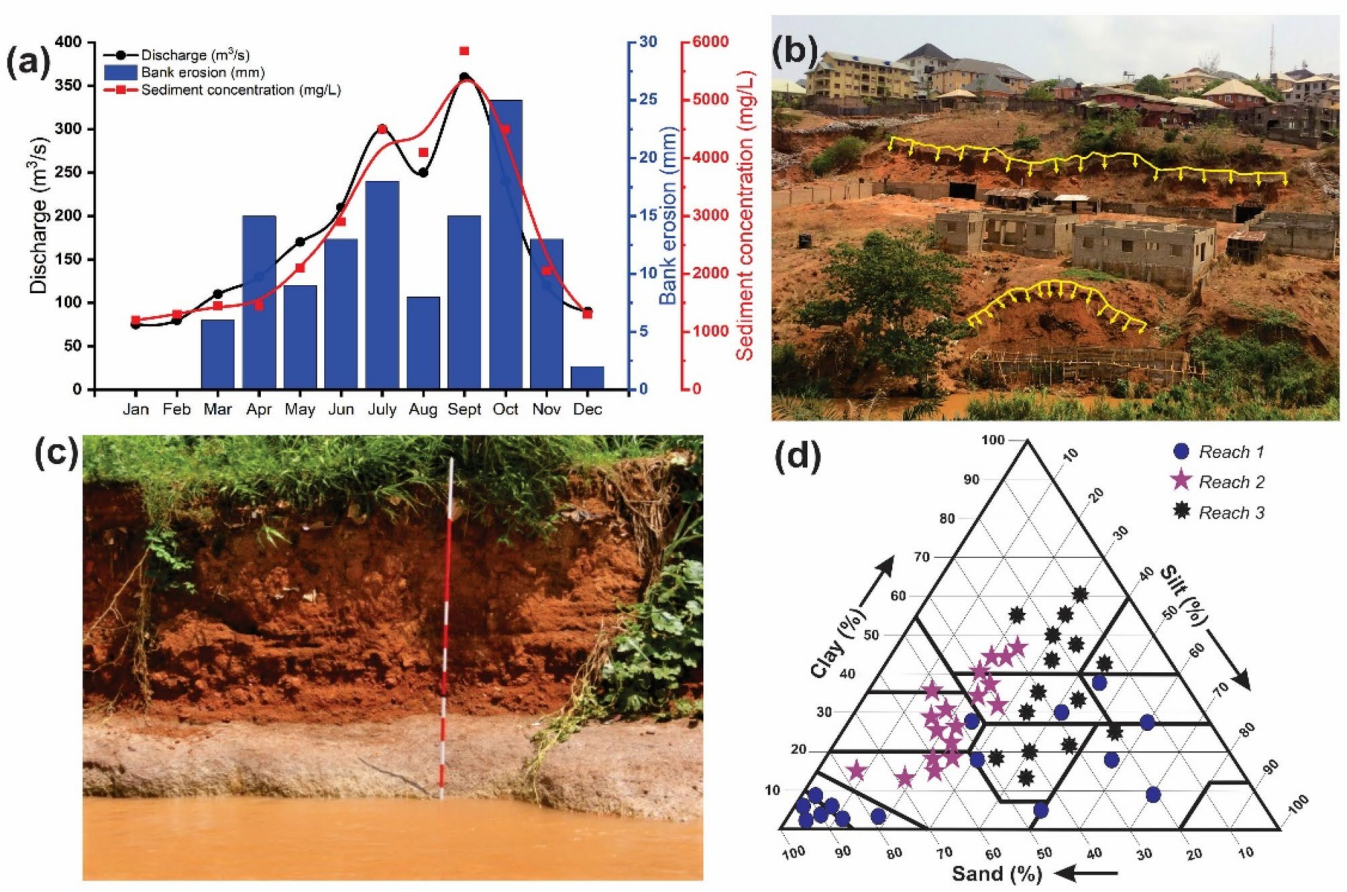
(**a**) Relationship among mean annual discharge, sediment concentration and bank erosion measured at *Reach 3* (**b**) Rotational failure of *Reach 1* streambanks with obvious slides and slump surfaces at the upper parts of the riparian area (**c**) Slab failure of the composite streambank at *Reach 3* (**d**) Ternary diagram of clay, silt and sand fractions from the soil samples recovered from streambanks in the three study reaches.

Bank erosion rates at the 21 study sites in the watershed varied across reaches due to the spatio-temporal differences in the hydrologic regime and geotechnical properties of the bank materials (Fig. 4). Bank erosion rates generally increased from the bank top to the bank toe in all the reaches except in *Reach 3* where the high erosion resistance of the underlying clay unit within the composite banks changed the erosion dynamics of the river channel. The results further indicate that erosion rates in *Reach 1* and *Reach 2* increased from 16.4 and 12.3 cm during the 2017–2018 monitoring period to 39.8 and 30.8 cm during the 2019–2020 monitoring period, respectively. Mean erosion rates of 12.2, 20.9 and 29.3 cm were recorded in all the reaches during the three monitoring periods: 2017–2018, 2018–2019, and 2019–2020, respectively (Table 5). Bank erosion rates and sediment concentration were significantly associated with extremely high flow events, as exemplified by the 2017–2018 mean monthly discharge and erosion pin data from *Reach 3* (Fig. 5a). Bank retreat generally occurred during the rainy season with the highest erosion rate of ~ 2.3 cm in October, while negligible retreat or small deposition of talus material at the bank toe occurred during the peak dry season months of December, January, and February. The majority of the riparian corridors where high bank erosion rates (> 30 cm) were recorded have been under active urban expansion or dominated by bare land and row crops. Furthermore, mean recession rates from the three reaches were 10.7 cm (2017–2018 period), 14.7 (2018–2019 period), and 17.5 cm (2019–2020 period). *Reach 1* streambanks had the highest recession rate (mean value, 17.2 cm) during the three-year monitoring period, while *Reach 3* banks recorded the lowest recession rate (mean value, 11.2 cm) during the same monitoring period. The overall average contribution from bank erosion (sediment loss) for the three reaches increased from 983 t/km during the 2017–2018 period to 1582 t/km during the 2019–2020 period. The highest average sediment loss was recorded in *Reach 1* (1751 t/km), while average sediment loss of 1479 and 670 t/km were recorded from *Reach 2* and *Reach 3*, respectively. The positive relationship between discharge and sediment concentration with bank erosion is indicative of fluvial-induced bank instability that leads to high sediment loads in the river (Fig. 5b-c). During the three-year monitoring period, average sediment loads exported from NRW were 4415, 3514 and 7422 t for *Reach 1*, *Reach 2*, and *Reach 3*, respectively (Table 5). During the three-year monitoring period, about 40% of the total annual sediment loads from *Reach 1* were ascribed to streambank erosion. Similarly, about 42 and 9% of the  total annual sediment loads from *Reach 2* and *Reach 3* were related to streambank erosion.

**Table 5:** Summary of average value per reach of bank height, mean annual discharge, erosion and recession rates, total suspended sediment load, and sediment export from the Nkisi River watershed.

Reach	Reach 1	Reach 2	Reach 3	Overall average
Mean bank height (m)	6	6	4	5.3
Mean annual discharge (m^3^/s)	104	84	177	122
**Bank erosion rate (cm)**				
2017–2018	16.4	12.3	7.9	12.2
2018–2019	30.4	20.4	12	20.9
2019–2020	39.8	30.8	17.2	29.3
**Recession rate (cm)**				
2017–2018	14.0	10.5	7.5	10.7
2018–2019	17.5	15.0	11.5	14.7
2019–2020	20.0	18.0	14.5	17.5
**Total suspended sediment load (t)**				
2017–2018	3922	3034	6869	4608
2018–2019	4294	3404	7434	5044
2019–2020	5028	4103	7964	5698
**Total contribution from bank erosion (t/km)**				
2017–2018	1428	1071	450	983
2018–2019	1785	1530	690	1335
2019–2020	2040	1836	870	1582
**Percentage of total watershed sediment export**				
2017–2018	36.4%	35.3%	6.6%	26.1%
2018–2019	41.6%	44.9%	9.3%	31.9%
2019–2020	40.6%	44.7%	10.9%	32.1%
**Major riparian land use**				
2017–2018	Grassland/cropland	Grassland/built-up	Grassland/cropland	
2018–2019	Cropland/built-up	Built-up/grassland	Cropland/built-up	
2019–2020	Built-up/bare land	Built-up/bare land	Built-up/cropland

A ternary diagram of the 47 soil cores recovered from the three reaches and plotted using the USDA Textural Soil Classification System reveals that more than 45% of *Reach 1* bank materials can be classified as sand and loamy sand (Fig. 5d). Furthermore, the majority of *Reach 2* bank materials can be classified as sandy clay loam, clay loam and clay, while *Reach 3* bank materials can be classified as loam, clay loam and clay. This variability in the textural characteristics of the bank materials can be attributed to the varying bank erosion rates in all the reaches and within the composite bank of *Reach 3*.

### Geotechnical characteristics of the bank materials

**Figure 6:**
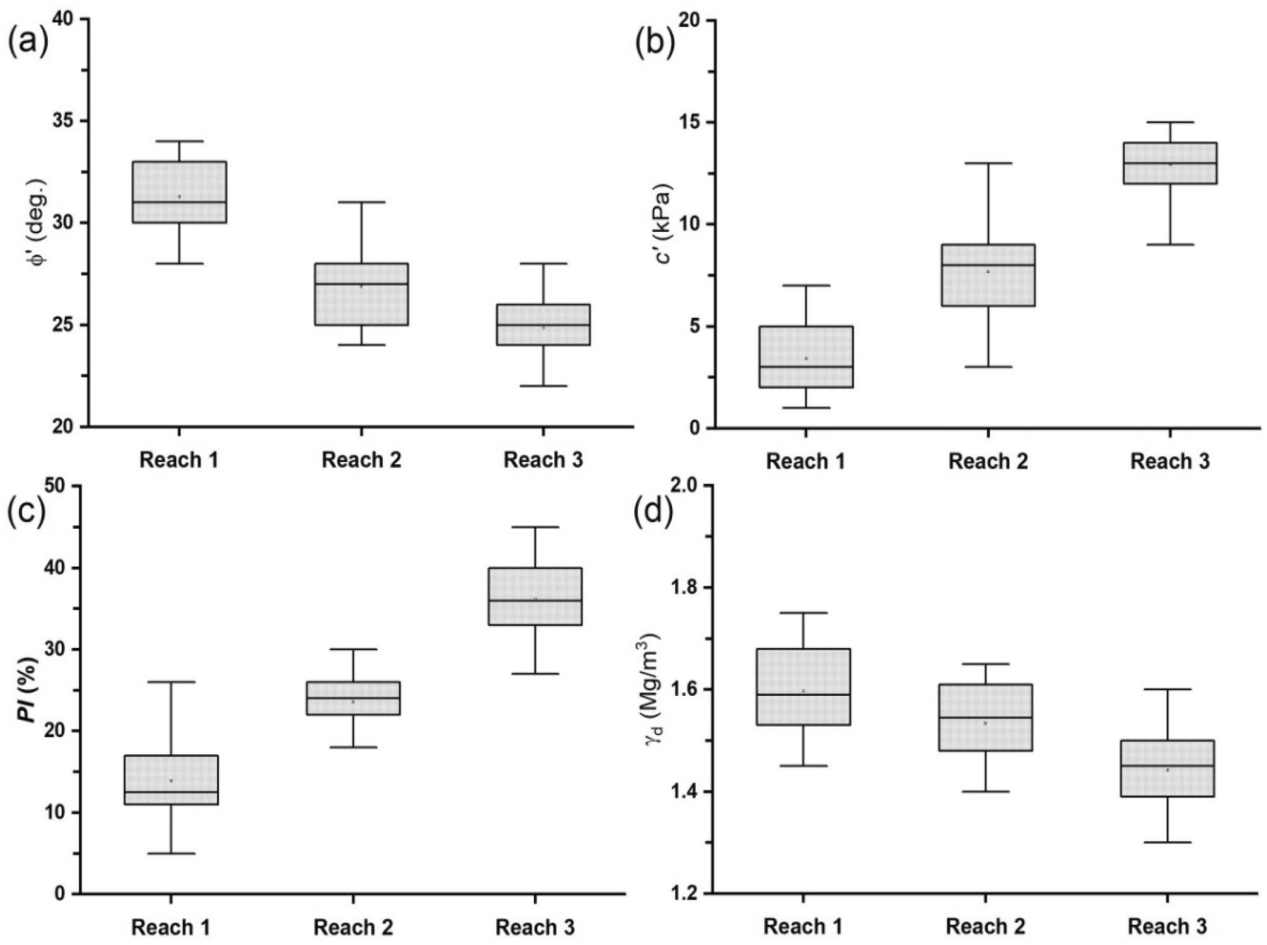
Box plots of (**a**) effective internal friction angle ($\varphi^{\prime}$) (**b**) effective cohesion ($c^{\prime}$) (**c**) plasticity index (*PI*), and (**d**) dry density ($\gamma_{d}$) from the streambank materials within the three study reaches.

The variation of the dominant geotechnical properties of the bank materials across the three reaches was evaluated by using box plots (Fig. 6). The results show that the dominant geotechnical properties of the bank materials varied across reaches. The values of effective internal friction angle ($\varphi^{\prime}$) obtained from a series of UU triaxial tests generally decreased from *Reach 1* to *Reach 3* with a mean value of 31.3°, 26.9°, and 24.9° for *Reach 1*, *Reach 2*, and *Reach 3*, respectively (Fig. 6a). In contrast, the effective cohesion values ($c^{\prime}$) increased from *Reach 1* to *Reach 3* with a mean value of 3.4, 7.7, and 12.9 kPa for *Reach 1*, *Reach 2*, and *Reach 3*, respectively (Fig. 6b). Similar trends of geotechnical characteristics were observed in the plots of plasticity index (*PI*) and dry density ($\gamma_{d}$). The minimum and maximum values of *PI* in the three reaches were 5 and 26%, 18 and 30%, and 27 and 45% for *Reach 1*, *Reach 2*, and *Reach 3*, respectively (Fig. 6c). The box plots of $\gamma_{d}$ for all the reaches were mostly symmetrical, suggesting that the mean is almost the same as the arithmetic mean. The mean values of $\gamma_{d}$ in all the reaches decreased from 1.60 Mg/m^3^ for *Reach 1* to 1.53 and 1.44 Mg/m^3^ for *Reach 2* and *Reach 3*, respectively (Fig. 6d). Based on the grain-size distribution and Atterberg limits test results, the bank materials in *Reach 1* can be classified as well-graded sand (SW), silty sand and clayey sand (SM-SC) following the Unified Soil Classification System (USCS). Also, *Reach 2* bank materials can be classified as SC, low plasticity silt (ML), inorganic clay of low plasticity (CL), and organic clay of low plasticity (OL), while *Reach 3* bank materials are essentially low plasticity silty clays (ML-CL) and inorganic clays and organic clays/silts of high plasticity (CH-OH). The variation in the dominant geotechnical properties of the bank materials could be attributed to the mean bank erosion rates determined from the three reaches during the three-year monitoring period^46^.

### Bank stability analysis

**Figure 7:**
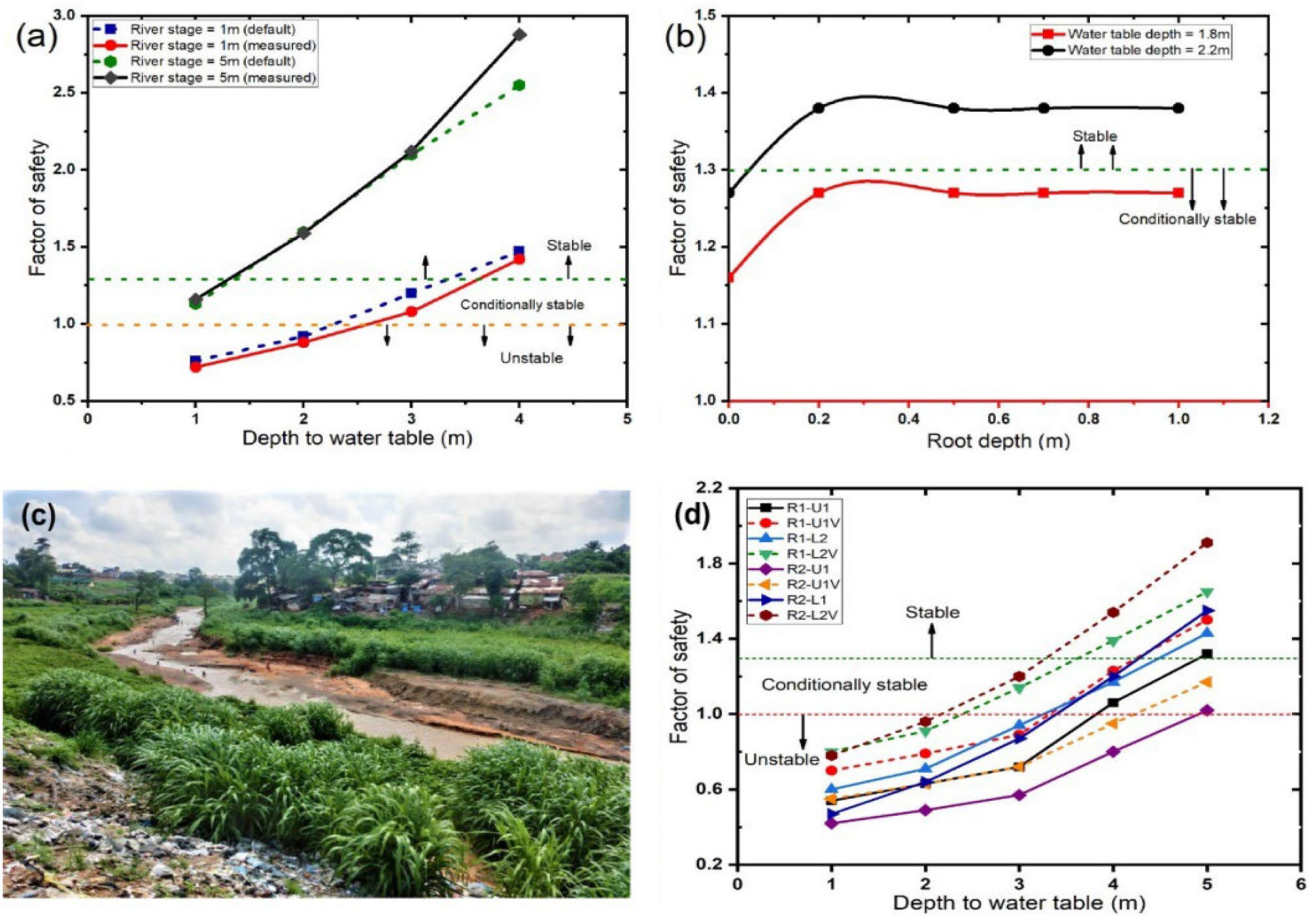
(**a**) Relationship between factor of safety and depth to groundwater level at river stages of 1 and 5 m based on the measured and default geotechnical properties of the 6-m streambanks at *Reach 1* and *Reach 2* (**b**) Relationship between factor of safety and rooting depth at depth to groundwater level of 1.8 and 2.2 m for the 4-m streambank at *Reach 3*. (**c**) Dominant vegetation cover of the riparian area at *Reach 3* showing the distribution of *Pennisetum purpureum*. (**d**) Relationship between factor of safety and depth to groundwater level for the 6-m streambanks at *Reach 1* and *Reach 2*. (*R1-U1* = unreinforced streambank at the upper part of *Reach 1*; *R1-U1V* = root-reinforced streambank at the upper part of *Reach 1*; *R1-L2* = unreinforced streambank at the lower part of *Reach 1*; *R2-U1* = unreinforced streambank at the upper part of *Reach 2*; *R2-U1V* = root-reinforced streambank at the upper part of *Reach 2*; *R2-L1* = unreinforced streambank at the lower part of *Reach 2*; *R2-L2V* = root-reinforced streambank at the lower part of *Reach 2*).

Figure 7a shows the relationship between $GWT_{depth}$ and $FoS$ for the 6-m composite banks at *Reach 1* and *Reach 2*. The simulations were carried out by considering two stream stage scenarios (1 and 5 m), which represent the mean flow elevation of the stream during the dry and rainy (wet) seasons, respectively. Also, the degree of accuracy of the simulation results was assessed by conducting the stability analysis using the default geotechnical parameters of the composite banks in the BSTEM and the results obtained from laboratory tests. The bank stability analysis results show that the stream stage relative to the $GWT_{depth}$ has a significant effect on the rate and magnitude of bank erosion and sediment loading, both for the measured and default parameters. The $FoS$ values obtained with the default BSTEM geotechnical parameters and the measured geotechnical properties of the bank material were approximately the same and therefore confirmed the validity of the input geotechnical parameters. At low stream stage scenarios (1 m), $FoS$ increased with a decrease in $GWT_{depth}$ and thus can be attributed to the increase in matric suction due to the dissipation of pore-water pressure that occurs during the falling limb of the stream stage hydrograph under rapid drawdown conditions^20,21^. The lowest degree of bank stability occurred at $GWT_{depth}$ of 1 and 2 m (unstable, $FoS \le 1$) due to the relatively steep hydraulic gradient of the water table relative to the stream stage. This occurs under rapid drawdown conditions, coupled with the fluvial undercutting of the bank toe and geotechnical failure of the moderately consolidated middle to upper sections of the bank faces. Results of numerous field, laboratory and numerical modelling investigations also demonstrated that the rate and magnitude of bank erosion and sediment loading were usually high during the recession stages of stormflow hydrographs under rapid drawdown conditions^47,48^.

Figure 7b shows the relationship between $FoS$ and plant rooting depth for the 4-m composite bank at *Reach 3*, considering $GWT_{depth}$ of 1.8 and 2.2 m, respectively. The result indicates that higher values of $FoS$ were obtained at $GWT_{depth}$ of 2.2 m relative to the stream stage of 1 m, whereas lower values of $FoS$ were obtained when the $GWT_{depth}$ increased from 2.2 to 1.8 m. The addition of 0.2 m rooting depth (wet meadow vegetation) to the bank produced $c_{r}$ values of 17.55 and 17.66 kPa, which increased $FoS$ from 1.16 and 1.26 to 1.27 and 1.38 at $GWT_{depth}$ of 1.8 and 2.2 m, respectively. One major factor that may account for the high $FoS$ obtained at $GWT_{depth}$ of 2.2 m is the low riparian hydraulic gradient due to baseflow recession^49^. Further increase in rooting depth beyond 0.2 m resulted in a decrease in $c_{r}$ and caused no further increase in $FoS$, irrespective of the river stage. Similar research by Abernethy and Rutherfurd^25^ reported that the $c_{r}$ value of most riparian forest buffers decreases with depth and is a function of the root area ratio.

Given the widespread distribution of *Pennisetum purpureum* (elephant grass) along the riparian corridors, especially at *Reach 3* (Fig. 7c), bank stability analysis was carried out to assess their influence on the stability of four of the most actively eroding streambanks at *Reach 1* and *Reach 2*, which contribute more than 30% of total sediment loads to the stream. The stability analysis made use of the default mechanical properties of *Tripsacum dactyloides* (eastern gamma grass) available in the RipRoot database of the BSTEM because it shares similar root density with *Pennisetum purpureum*. The stability analysis was carried out under rapid drawdown conditions by varying $GWT_{depth}$ from 1 to 5 m. The streambanks are characterised by oversteepened slopes that vary from 85° to 110°. The results indicate that the stability of the root-reinforced streambanks increased by 30% relative to the unreinforced banks, which were mostly unstable and conditionally stable (Fig. 7d).

## Discussion

### Factors and mechanisms controlling streambank erosion in NRW

The scale and magnitude of streambank erosion and accretion in many watersheds are controlled by the linkages between several influencing factors, such as climate, stream power, riparian land use, bank geometry, and lithology^50,51^. Therefore, the development of streambank erosion prevention and remediation measures requires a detailed understanding of its processes and triggering factors. Major insights into the conditioning factors that trigger streambank erosion in several ecoregions and landscapes have been gained, and these have led to the development of several process-based models to predict streambank stability and quantify annual streambank contributions to the total watershed sediment export^35,52,53^. However, a clear understanding of the linkages between bank erosion and sediment export in peri-urban watersheds remains the focus of geomorphologists, soil conservationists, researchers, town planners, and policymakers.

The NRW and its environs are affected by severe land degradation processes, including bank erosion/accretion, rill and gully erosion. Long-term monitoring and observation of the riparian area revealed marked variability in bank erosion rates due to the spatio-temporal variation of bank lithology, geotechnical properties, stream power, and riparian forest buffers. *Reach 1* and *Reach 2* streambanks are mostly homogeneous and are characterised by reddish-brown, ferruginous clayey sand and silt, which are moderately consolidated and highly erodible. In contrast, *Reach 3 area* is predominantly made up of composite streambanks that are characterised by a basal stiff, resistant clay layer and an upper layer of moderately- to well-consolidated silty clay. The dominant failure mechanism of *Reach 1* streambanks is rotational failure which changes to shallow slides and slumps in the upland areas where intense deforestation and urbanisation have led to increased runoff and soil erosion (*cf.* Fig. 5b). Similarly, slab failures observed in *Reach 3* streambanks have been attributed to the fluvial undercutting of the basal section of the upper reddish-brown, moderately consolidated layers of the composite bank (*cf.* Fig. 5c). This marked difference in the erodibility of the bank materials within the three reaches could be ascribed to their different lithologic and sedimentological properties. Also, the differing lithologic and geotechnical properties of the streambanks result in variable erosion rates in the three study reaches. Furthermore, the scale and magnitude of bank erosion within the NRW are strongly influenced by the shear strength parameters of the bank materials, the distribution of riparian forest buffer, and to a lesser degree by bank geometry. The observed failure mechanisms of the streambanks can be divided into three (3) stages. *Stage I* involves subaerial processes, including desiccation cracking that occurs during the dry to early rainy season months of March and April when the stream stage and depth to groundwater level are significantly low. Subaerial processes reduce the shear strength of the soil and constitute the predisposing conditions that lead to fluvial erosion^22^. *Stage II* involves fluvial erosion processes that destabilise the streambank by removing in situ and accreted material at the bank toe. This stage is also associated with an increase in the specific weight of the soil, generation of positive pore-water pressures under high-intensity rainfall events, and reduction of shear strength of the soil leading to failure^20,21^. Rapid drawdown conditions generally trigger bank failure during the recession limb of the stream hydrograph. *Stage III* involves mass failure of overhanging blocks of material, leading to degradation and channel widening. Streambed lowering and accretion occur in some of the stream reaches where the stream power is too small to entrain the coarse-grained soil particles downstream and thus results in flood inundations onto adjacent floodplains. Consequently, numerous cases of building collapse and flood hazards have been recorded within the study area due to unregulated urban sprawl within the riparian corridors. Similar cases of bank erosion, accretion, channel migration, and associated hazards have been studied in detail using very high-resolution satellite images and GIS^30,54–56^.

### Role of riparian forest buffers on streambank stability

Riparian forest buffers play a significant role in climate change adaptation and mitigation. Hence any alteration in their natural disturbance regimes could potentially trigger several geomorphological processes, such as irregular hydrologic regimes, cyclic sequence of erosion and deposition, and bankline migration. Riparian forest buffers provide essential ecosystem services, such as cycling of nutrients, stabilisation of streambanks, restoration of aquatic and terrestrial habitats, trapping of sediments, and reduction of flood hazards, among others^27,57–60^. Therefore, riparian forest buffers are one of the major nature-based solutions (NbS) used to stabilise streambanks and restore the riparian ecosystem.

Results of this study clearly indicate that several anthropogenic activities, such as agricultural intensification, deforestation, and urbanisation, had an adverse impact on streambank stability and riparian ecosystem health^18,19^. The increase in urban centres from 2013 to 2019, at the expense of vegetation, had a very negative impact on the stability of streambanks within the study area, as evidenced by the total contribution from bank erosion, which increased from 983 to 1582 t/km during the 2017–2020 monitoring period. Similar results have been reported by Laubel et al.^61^, Veihe et al.^62^, and Kronvang et al.^63^ in their research to quantify the rates and magnitude of bank erosion and streambank sediment delivery to several streams of Denmark using erosion pins and laboratory analysis. More than 95% of streambank erosion cases within *Reach 1* and *Reach 2* can be attributed to the rapid destruction of the riparian forest buffer^48,57^. It is important to note that the rate of accretion/sedimentation at the river mouth is directly attributed to the progressive destruction of the riparian forest buffers due to urbanisation and other anthropogenic activities that lead to bank erosion and channel planform changes. Hence, this research supports previous findings by Keesstra et al.^10^, Bernier et al.^31^, and Rajakumari et al.^64^ that urbanisation, deforestation, agricultural intensification, and other human-induced disturbances in the riparian ecosystem are the major factors controlling the frequency and magnitude of bank erosion, sediment supply, and changes in channel planforms in many urban and peri-urban landscapes.

Furthermore, this research has been able to demonstrate the positive influence of riparian vegetation on streambank stability in the study area, as supported by previous studies carried out by Pollen and Simon^42^, Simon and Collison^27^, Wynn and Mostaghimi^22^, and Yu et al.^29^. The present study provides new insights into the relationship between factor of safety and plant rooting depth under varying depths to groundwater level and stream stage and thus shows that depth to groundwater level has a significant effect on streambank stability. A similar bank stability analysis carried out by Zegeye et al.^65^ at a gully erosion site in the Ethiopian highlands showed that gully banks reinforced with plant roots have a higher factor of safety than unreinforced gully banks. The authors further observed that the stability of the root-reinforced gully banks depends on the depth to groundwater level and bank slope angle. Based on the current results, it can be observed that the 6-m streambanks reinforced with *Pennisetum purpureum* are prone to failure, especially during high stormflow events when the depth to groundwater level rises due to the inflow of water into the riparian slope. Therefore, reinforcing the streambanks with *Pennisetum purpureum* and other native shrubs in addition to protecting the bank toe with large woody debris, plant cuttings and rip raps could minimise the hydraulic forces of the river and prevent the erosion of the streambanks. Also, the factor of safety of the streambanks could be increased by introducing native grasses and shrubs of deep root systems and by planting drought-resistant grasses on the highly erodible bank faces to anchor the soil particles in place and minimise fluvial erosion and undercutting of the bank toe, especially during high stream stage scenarios. Lastly, the placement of large woody debris along the stream corridors following the design method adopted by Shields et al.^66^ could inadvertently lead to sediment retention and prevent further recession of the streambanks while improving the overall health of the riparian ecosystem.

## Conclusions

This study utilised multi-temporal datasets acquired from remotely sensed Landsat images to evaluate the dominant land use classes contributing to streambank erosion and accretion in a small, peri-urban watershed of southeast Nigeria. GIS and RS data were integrated with in situ bank erosion measurements and bank stability analysis to evaluate the recession rates of the streambanks and determine the appropriate ecohydrological and hydro-geotechnical solutions that would stabilise the riparian corridors and restore the riparian ecosystem. Bank erosion and accretion in the NRW depend solely on the spatiotemporal variability of vegetation and land use. Built-up and sand deposit/floodplain increased from 2003 to 2019 at the expense of vegetation and water body. Thus, the high supply of sediment from bank erosion and overland flow processes led to high accretion rates at the river mouth. Bank erosion rates varied in all the reaches, with high erosion rates in riparian corridors dominated by cropland, built-up and bare land. The total contribution from bank erosion increased in all the reaches from the 2017–2018 monitoring period to the 2019–2020 monitoring period, with *Reach 1* and *Reach 3* recording total average values of 1751 and 670 t/km of sediment, respectively. The recession rates of the streambanks and the total suspended sediment loads increased throughout the monitoring period, while bank erosion contributed up to 40.6% and 44.7% of total sediment exported from the watershed in *Reach 1* and *Reach 2*, respectively, during the 2019–2020 monitoring period. Streambank failures in all the study sites were mostly attributed to fluvial erosion and, to a lesser degree, geotechnical failure, irrespective of their differing geotechnical and lithologic characteristics. The failure sequence of the streambanks in *Reach 1* and *Reach 2* were dominated by (1) the initial undercutting of the bank toe by the hydraulic forces of the flowing water, especially during the peak flow stages of the river hydrograph and (2) geotechnical failure of the unstable, upper parts of the bank. The BSTEM results indicated that plant roots have a mechanical effect on riparian slopes. However, the mechanical effect of plant roots on bank stability tends to diminish under rising groundwater table, especially during high streamflow events. *Tripsacum dactyloides* increased the factor of safety of the streambanks by 30%; therefore, planting *Pennisetum purpureum* and other native shrubs of high root density along the riparian slopes would likely increase the stability of the streambanks and lead to the restoration of the riparian ecosystem.

Our results highlight the significance of monitoring watershed-scale sediment yield and export under different land uses to determine channel responses to diverse disturbance regimes. The results also demonstrate the importance of integrating traditional field surveys and process-based numerical models to accurately determine the recession rates of streambanks. The proportion of total watershed sediment export ascribed to bank erosion is expected to increase as the entire watershed changes from a peri-urban to an urban landscape.

One of the major limitations of this research is the difficulty in quantifying the amount of watershed sediment export attributed to overland flow processes (surface runoff), especially where the riparian land use is characterised by cropland, bare land or built-up. The contribution of surface runoff into the stream channels oftentimes increases the stream load, which inadvertently creates some disparities in the total watershed sediment export attributed to bank erosion. Also, the bank stability analysis results performed with the BSTEM are limited by the fact that the default parameters in the root-reinforcement submodel were used to evaluate the influence of vegetation on the stability of streambanks. While empirical evidence suggests that the rate of erosion, deposition and accretion were directly related to the LULC dynamics in the Nkisi River watershed, causality tests need to be performed to establish this relationship. Furthermore, future work on the study area should be done with precision monitoring tools such as the Photo-Electronic Erosion Pins (PEEPs), with smaller spacing between pins, together with terrestrial laser scanners, to accurately determine streambank erosion rates in the study area. Finally, we recommend utilising sustainable nature-based solutions and other soft (ecological) engineering techniques to increase the stability of the riparian slopes and improve the overall ecological health of the riparian corridors.

## Supplementary Information

Supplementary Information.

## Data Availability

The datasets used and/or analysed during the current study are available from the corresponding author on reasonable request.
