# Supplementary material for: An integrated assessment of land use impact, riparian vegetation and lithologic variation on streambank stability in a peri-urban watershed (Nigeria)
Source: Sci Rep. 2022 Jun 29;12:10989. doi: 10.1038/s41598-022-15008-w (PMC9243088; doi:10.1038/s41598-022-15008-w)
Supplement: Supplementary file 1 — Supplementary Information. [file 41598_2022_15008_MOESM1_ESM.docx]

**SUPPLEMENTARY DATA**

Link to the source files of the land use/ land cover classification maps of the study area: https://drive.google.com/folderview?id=1cKZGbSTTD-XWimYpO68c-DCb3oznXlKU
